# The role of local heating in the 2015 Indian Heat Wave

**DOI:** 10.1038/s41598-017-07956-5

**Published:** 2017-08-09

**Authors:** Debjani Ghatak, Benjamin Zaitchik, Christopher Hain, Martha Anderson

**Affiliations:** 1 Department of Earth and Planetary Sciences, Johns Hopkins University, Baltimore, Maryland USA; 2 Department of Earth and Planetary Sciences, Johns Hopkins University, Baltimore, Maryland USA; 3 NASA Marshall Space Flight Center, Earth Science Branch, Huntsville, Alabama USA; 4 Hydrology and Remote Sensing Laboratory, Agricultural Research Service, USDA, Beltsville, Maryland USA

## Abstract

India faced a major heat wave during the summer of 2015. Temperature anomalies peaked in the dry period before the onset of the summer monsoon, suggesting that local land-atmosphere feedbacks involving desiccated soils and vegetation might have played a role in driving the heat extreme. Upon examination of *in situ* data, reanalysis, satellite observations, and land surface models, we find that the heat wave included two distinct peaks: one in late May, and a second in early June. During the first peak we find that clear skies led to a positive net radiation anomaly at the surface, but there is no significant sensible heat flux anomaly within the core of the heat wave affected region. By the time of the second peak, however, soil moisture had dropped to anomalously low levels in the core heat wave region, net surface radiation was anomalously high, and a significant positive sensible heat flux anomaly developed. This led to a substantial local forcing on air temperature that contributed to the intensity of the event. The analysis indicates that the highly agricultural landscape of North and Central India can reinforce heat extremes under dry conditions.

## Introduction

Uncomfortably high temperatures are an expected condition in India during the weeks prior to onset of the monsoon. The climatological average temperature for the month of May is above 35 °C in large parts of north and Central India, making it the hottest month in the calendar over North India. Nevertheless, some years stand out for their extreme heat, including 1998^1^, 2003^2^, 2005^3^ and both 2015 and 2016. The heat wave of 2015 (HW15) received significant coverage in the international media, as it had dramatic impacts on large population centers and has been blamed for more than 2500 human deaths^4^.

The impacts of recent heat waves are of particular concern since these events are expected to become more frequent, intense, and of longer duration for much of India over the course of the 21^st^ century^5^. The fact that extreme heat events tend to come just before the onset of monsoon rains also raises an interesting question about land-atmosphere interactions. This is a dry time of year in much of India, and both the approach of summer solstice and the presence of typically clear skies lead to high downwelling solar radiation at the surface. This suggests that extreme heat waves could, in part, be a product of local heating through enhanced sensible heat flux from a hot and dry surface. A significant contribution of local heating to the onset and/or intensification of heat waves has been found for major heat events in Europe in 2003^6, 7^ and in Russia in 2010^8^, among others. Impacts of depleted soil moisture on the occurence of heat wave during 1961–2013 are also found over India^9^. Anecdotally, extreme heat events appear to be associated with late monsoon rains, inadequate pre-monsoon rains, or low rain in neighboring regions leading to advection of dry heat into India^10^. Longer (duration) and warmer heat waves over India are found to be linked with El Niño years as well^11^.

Here we perform a detailed investigation of HW15, that is designed to: (1) define the temporal and spatial pattern of the event, since media reports of impacts do not necessarily align with the actual climate anomaly; and (2) characterize the role that surface conditions—in particular, soil moisture anomaly and associated sensible heat flux anomalies play in the onset and evolution of the event. This diagnostic analysis of land-atmosphere processes complements recent studies of the predictability of HW15^4^ and its connection to large scale atmospheric circulations^10^.

## Results and Discussion

### Description of the heat wave

**Figure 1:**
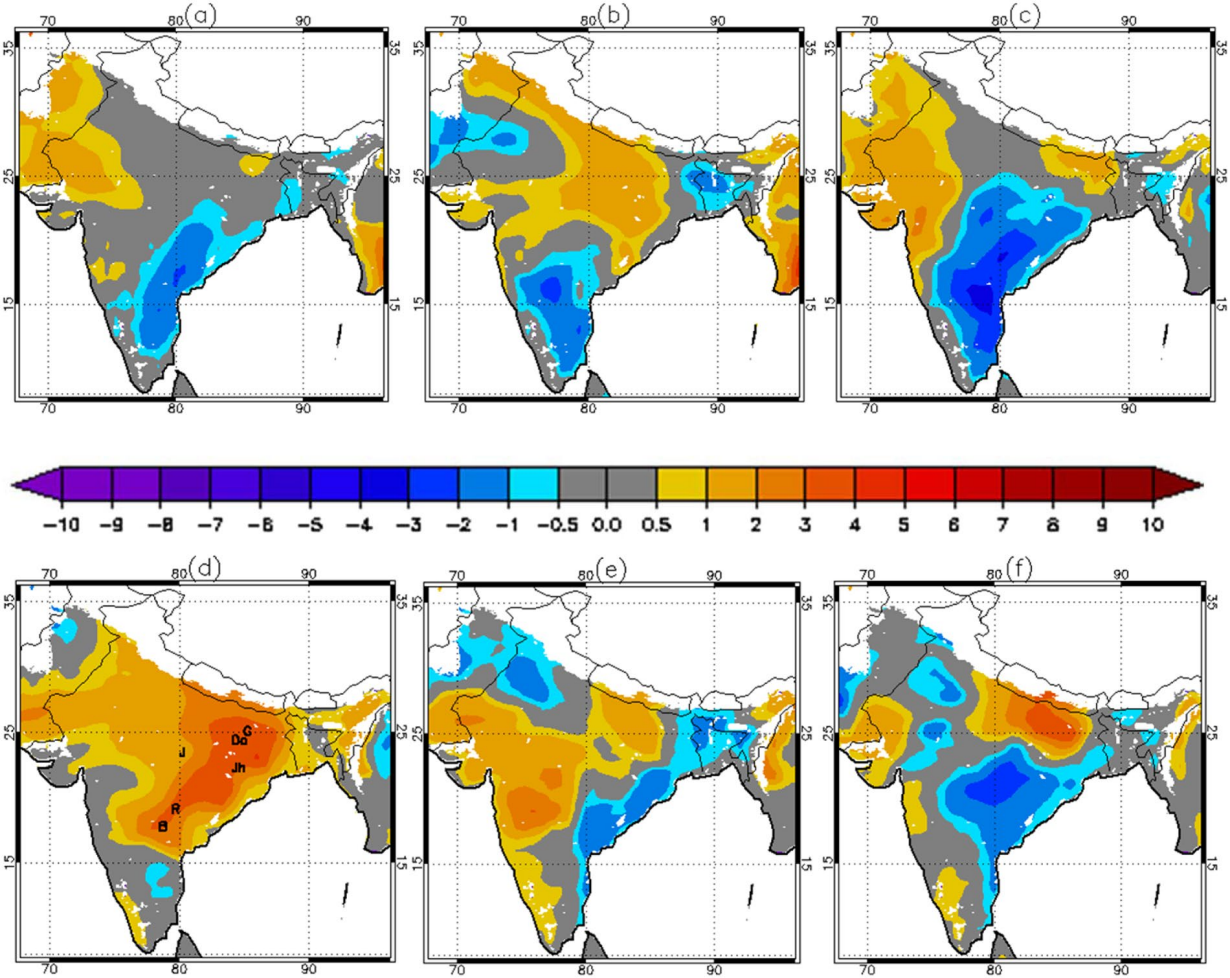
Weekly anomaly of SAT (°C) for the weeks of (**a**) April 30^th^–May 1^st^ to May 6^th^−7^th^, (**b**) May 7^th^−8^th^ to May 13^th^−14^th^, (**c**) May 14^th^−15^th^ to May 20^th^−21^st^, (**d**) May 21^st^−22^nd^ to May 27^th^−28^th^, (**e**) May 28^th^−29^th^ to June 3^rd^−4^th^ and (**f**) June 4^th^−5^th^ to June 10^th^−11^th^ in 2015 based on the weekly climatology of 1980–2015. Station locations (G = Goya, Da = Daltonganj, Jh = Jharsuguda, J = Jabalpur, R = Ramgundam and B = Begumpet Airport) are marked in Fig. 1d. Any pixel with elevation above 1000 m is not shown (white colored region). Data visualizations produced using IDL [8.4] (Exelis Visual Information Solutions, Boulder, Colorado).

We define the temporal and spatial domain of HW15 in terms of anomaly thresholds in the rolling seven day (one week) average surface air temperature (SAT). This is just one of many ways to define a heat wave event. We choose this approach because the prolonged persistence of elevated temperature was a defining feature of HW15. Anomalies were calculated on a gridcell by gridcell basis relative to 1980–2015 climatology using MERRA-Land (MLD) SAT estimates (Fig. 1). Very high weekly SAT anomalies are apparent in both late May (May 21st-22nd to May 27th-28th) and early June (June 4th-5th to June 10th-11th) (Fig. 1). On this basis, we define the Core of the Heat Wave (COHW) region for both the late May (COHW_May_) and early June (COHW_June_) peaks as the region within India in which the weekly SAT anomaly exceeded 3 °C. Both COHW are located in the eastern half of India. However, COHW_May_ is large and extends over south India, while COHW_June_ is smaller and is focused in the north of the Gangetic Plain. A statistically-defined threshold, where pixels meeting or exceeding the 90th percentile threshold weekly SAT for rolling seven day average SAT are defined as being in heat wave status yielded similar results for the late May peak (See Supplementary Fig. S1). The 3 °C absolute anomaly threshold was slightly more spatially coherent than the 90th percentile threshold and was used as the basis for further analysis.

**Figure 2:**
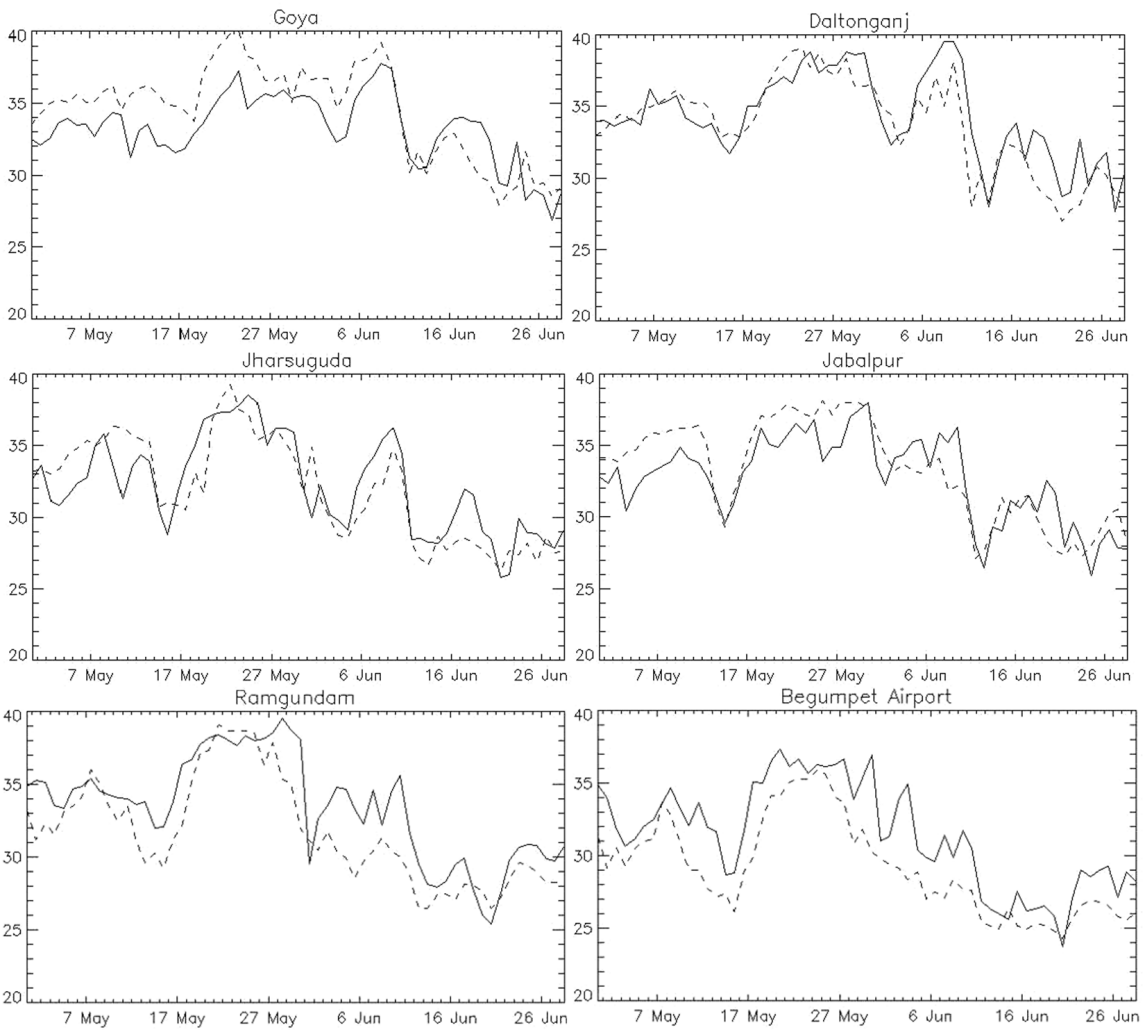
Daily SAT (°C) during May and June in 2015 for station observations (solid line) and MLD output (dashed line). Data visualizations produced using IDL [8.4] (Exelis Visual Information Solutions, Boulder, Colorado).

Synoptic weather station records which are geographically located in and around COHW_May_ are shown in Figs 2 and S2. The box and whisker diagram (Fig. S2) shows the comparison between daily SAT from observations and from MLD for a long-term record. The match between MLD and stations is not perfect, but the general pattern holds and correlation between MLD and station SAT is high for all selected stations (Supplementary Tables S1 and S2). Figure 2 clearly show the consistent time domain from late May and early June in 2015, when very high SAT is observed at these stations. The two northernmost stations (Goya and Daltonganj) have highest temperature in June while the others peak in late May. Consequently, some stations show that there are two distinct temperature peaks: the first in late May, and the second in early June. These two peaks are separated by a period of elevated but not extreme temperatures. The two peaks evident in station data are also present in MLD SAT estimates (Fig. 2; dashed line). Notably, in terms of both absolute magnitude and deviation from the mean, the week of May 21st–22nd to May 27th–28th stands out above any warm conditions experienced earlier in the month (Figs 1 and 2). This is relevant because the monthly temperature anomaly (See Supplementary Fig. S3) includes hotspots in both the East and West of the country, but weekly analysis shows that only the eastern hotspot is the product of a focused heat wave event. A coherent departure reemerges in the week of June 4th-5th to June 10th-11th during the secondary HW15 peak.

### Local heating anomaly

**Figure 3:**
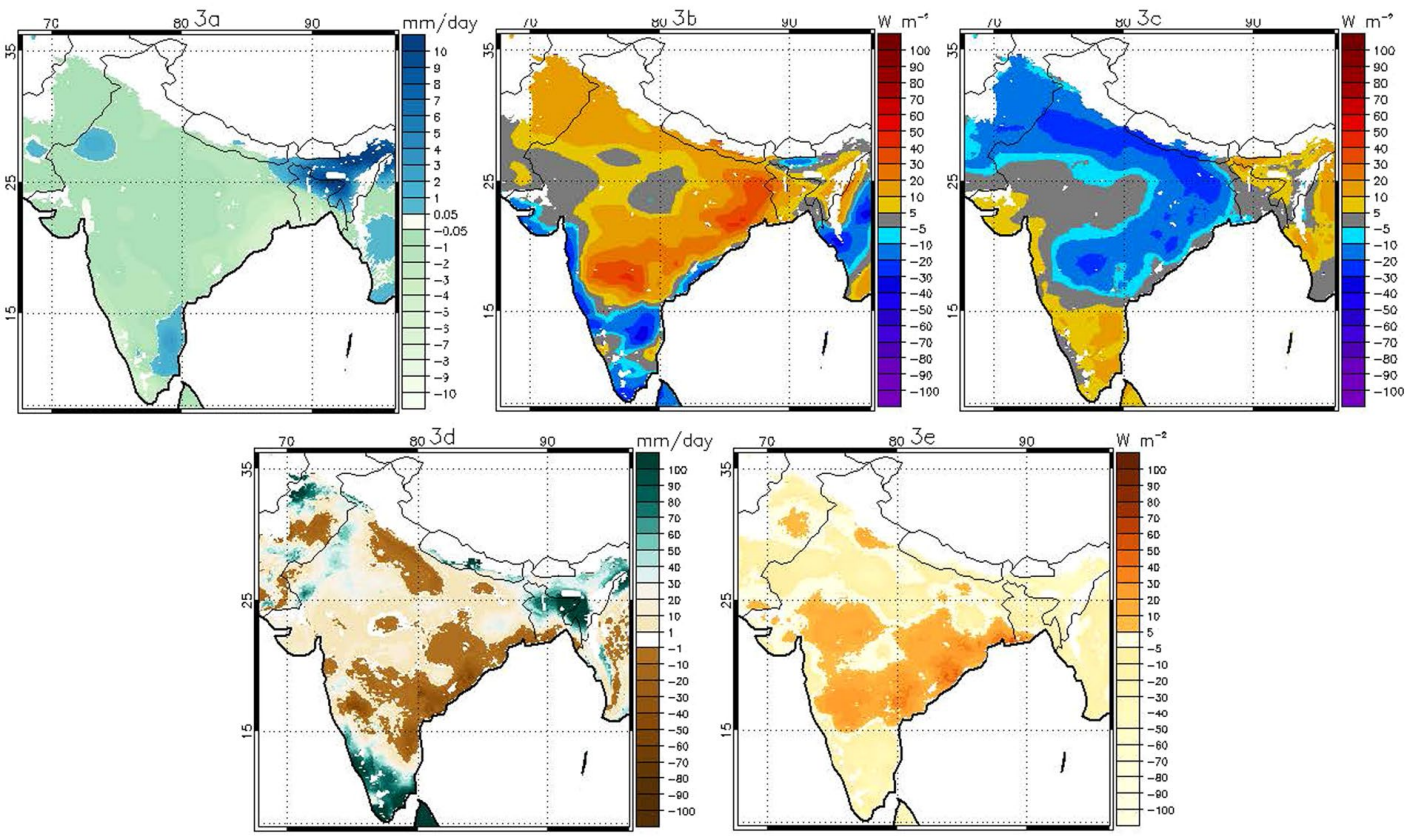
Anomalies of (**a**) total precipitation, (**b**) Net SW, (**c**) Net LW, (**d**) total profile soil moisture and (**e**) SH for the week of May 21^st^−22^nd^ to May 27^th^−28^th^ in 2015 based on the weekly climatology of 1980–2015. Any pixel with elevation above 1000 m is not shown (white colored region). Data visualizations produced using IDL [8.4] (Exelis Visual Information Solutions, Boulder, Colorado).

One possible explanation for the severity of HW15, and one that was noted in news reports at the time (https://en.wikipedia.org/wiki/2015_Indian_heat_wave), is that the heat was associated with poor rainfall conditions. Low rainfall conditions could lead to enhanced surface heat flux both due to positive radiative heating anomalies (increased upwelling surface longwave radiation) and increases in sensible heat flux (SH) resulting from high net surface radiation (R_net_) under clear sky, sunny conditions and/or reduced soil moisture (SM) leading to lower evaporative fraction (EF). We find that the late May heat wave peak corresponded to a period of anomalously low rainfall and anomalously high surface net shortwave radiation (SW_net_) across much of India (Fig. 3a,b). This was associated with an anomalously low net longwave radiation (LW_net_) at the surface (Fig. 3c), which indicates enhanced radiative warming of the lower atmosphere by the surface.

**Figure 4:**
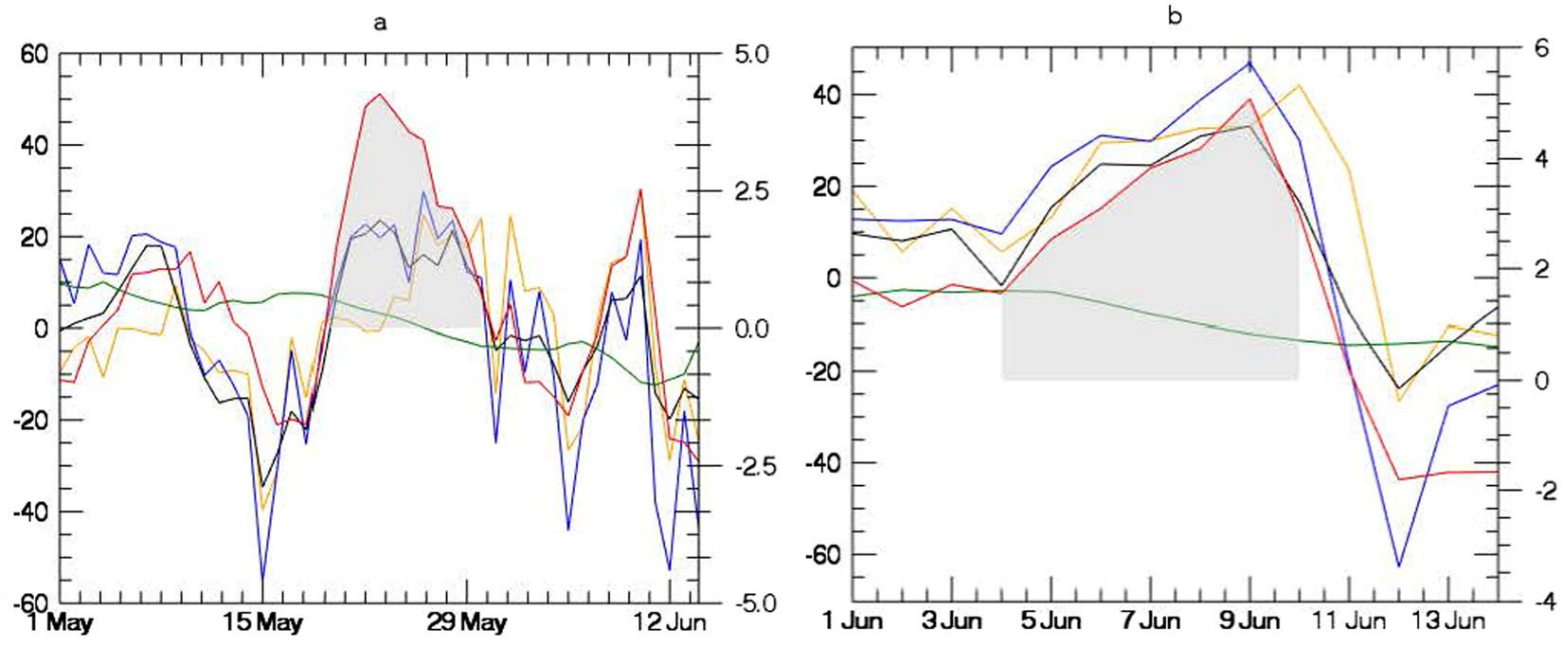
Average daily anomaly based on 1980–2015 daily climatology for a) COHW_May_ and for (**b**) COHW_June_ over Indian landmasses. The y axis scale on the right indicates SAT (°C, red line). The y axis on the left indicates sensible heat flux (W m^−2^, orange line), net SW radiation (W m^−2^, blue line), net LW radiation (W m^−2^, black line) and total profile soil moisture (mm, green line). Net LW radiation has been plotted with the reversed sign. Grey color shows the anomalously positive SAT during two extreme heat events during May and June. Data visualizations produced using IDL [8.4] (Exelis Visual Information Solutions, Boulder, Colorado).

The SM anomaly during this period, however, is mixed: southern portions of COHW_May_ show dry conditions (negative anomaly), but to the north soils are relatively wet (positive anomaly) (Fig. 3d). Following this SM pattern, the SH anomaly is also spatially variable, with a region of anomalously enhanced SH flux in the south of COHW_May_ that is larger than 30 Wm^−2^ in places, but areas of negative SH anomaly of similar magnitude to the north (Fig. 3e). Averaged across COHW_May_, we see that the May heat wave peaked during a period when the average SM anomaly was still positive (2.13 mm/day) and average SH anomaly was negligible (8 Wm^−2^) (Fig. 4 and Table 1). Only the SW_net_ anomaly was consistently positive and LW_net_ was consistently negative during this period, with average surface SW_net_ and LW_net_ anomalies on the order of 20.6 Wm^−2^ and −18.2 Wm^−2^ (Table 1).

**Table 1:** Average anomaly of the variables over COHW.

	Average for the period of May 21st-22nd to May 27th-28th in 2015 over COHW_May_	Average for the period of June 4th-5th to 10th-11th in 2015 over COHW_June_
SAT′	3.5 °C	3.32 °C
Net SW′	20.6 W/m^2^	30.1 W/m^2^
Net LW′	−18.2 W/m^2^	−20.5 W/m^2^
R_net_′	2.4 W/m^2^	9.56 W/m^2^
SM′	2.13 mm/day	−7.8 mm/day
SH′	8 W/m^2^	26.6 W/m^2^
Evaporative Fraction′	−0.08	−0.18
Potential SH contribution to heating	0.27 °C/day	0.9 °C/day
Mean near-surface wind speed	4.9 m/sec	4.5 m/sec

**Figure 5:**
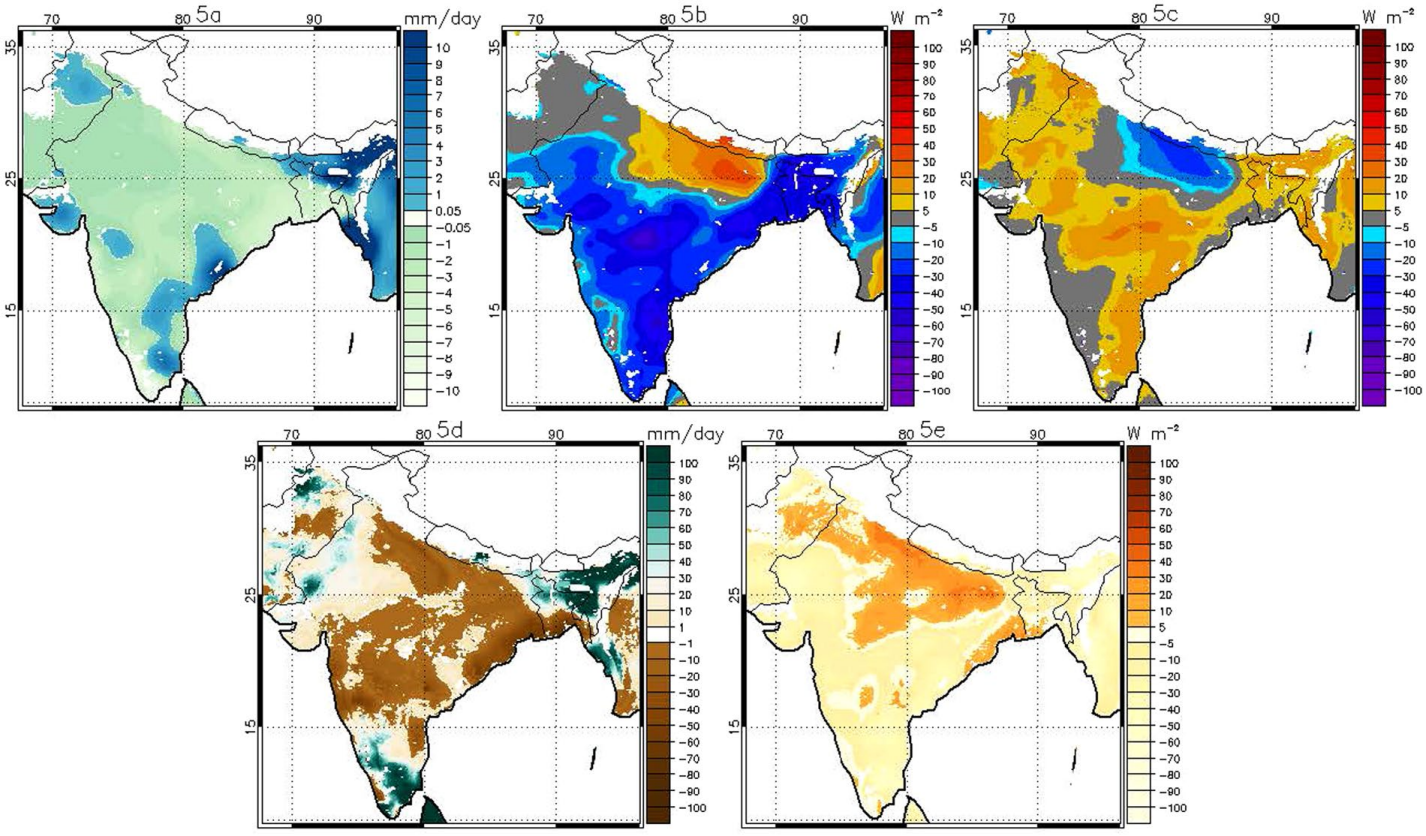
As in Fig. 3, but for the week of June 4^th^−5^th^ to June 10^th^−11^th^ in 2015. Data visualizations produced using IDL [8.4] (Exelis Visual Information Solutions, Boulder, Colorado).

In contrast to the May peak of the heat wave, the June peak occurred after the intense heat of May had dried the surface and as dry atmospheric conditions continued to prevail over northern India (Fig. 5a) where COHW_June_ is centered and localised. Therefore, the COHW_June_ is spatially much smaller than COHW_May_. Part of COHW_May_ (mainly the southern part of India) was spared from the June phase of the heat wave due to anomalously high rainfall over some areas (Fig. 5a); hence both *R*_*net*_ local forcing and SH local forcing were absent from the southern part of India. This second heat wave peak is characterized by negative SM anomalies across most of the heat-affected region (Fig. 5d) and enhanced SH anomaly across COHW_June_ (Fig. 5e). For this heat event, then, both the *R*_*net*_ local forcing and SH local forcing were active (Table 1): the surface LW_net_ anomaly was −20.5 W m^−2^ and the SH anomaly was 26.6 W m^−2^, across COHW_June_ (Table 1). The contrasts between the May and June peaks indicate that the May event was primarily a product of large scale forcings, including clear sky conditions that led to a local radiation feedback during the heat wave. The June peak, in contrast, emerged during a period of dry surface conditions and was characterized by large *SH* anomaly throughout the event.

**Figure 6:**
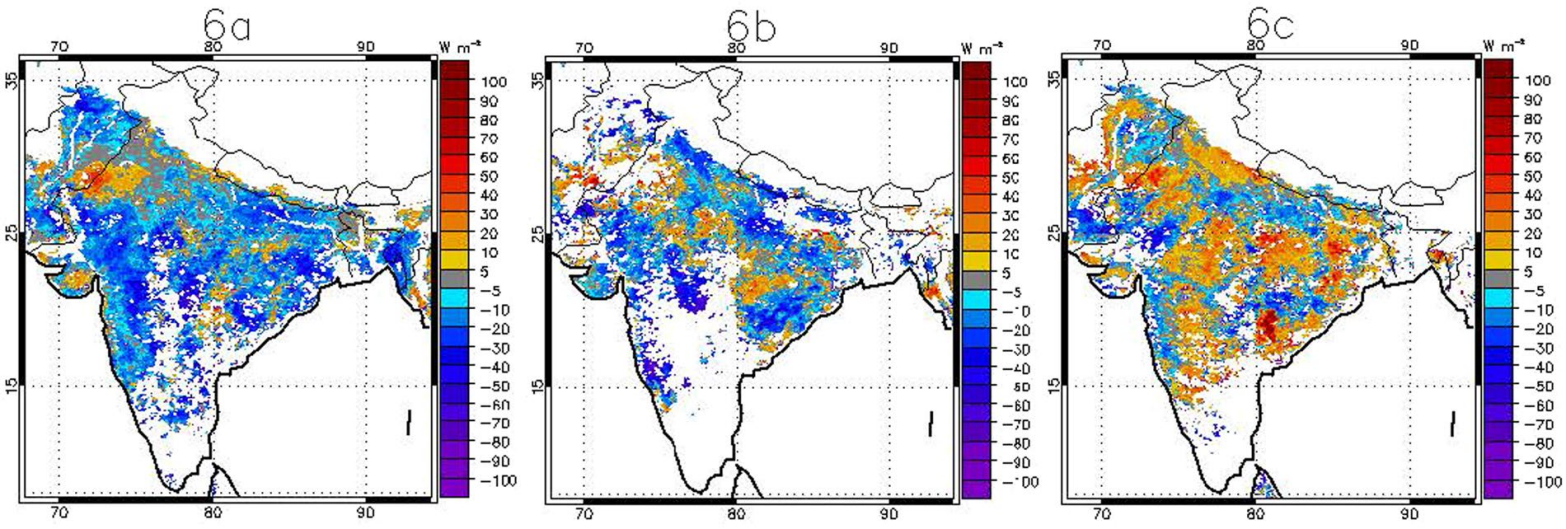
Sensible heat flux anomaly for the weeks of (**a**) May 7^th^−13^th^, (**b**) May 14^th^−20^th^ and (**c**) May 28^th^–June 4^th^ from ALEXI (see text for details of the dataset) based on the weekly climatology of 2003–2015. The anomaly plot for the weeks of May 21^st^−27^th^ and June 5^th^−11^th^ are not shown due to the large extent of missing data resulting from cloud-contamination in satellite inputs to ALEXI. ALEXI provides 7-day composite data where calendar dates for each of the 7-day periods are pre-defined. Any pixel with elevation above 1000 m is not shown (white colored region). White color also shows pixel with missing values. Data visualizations produced using IDL [8.4] (Exelis Visual Information Solutions, Boulder, Colorado).

The sequencing of these anomalies in MLD and in the LSM forced with MLD meteorology is confirmed by independent satellite estimates of sensible heat flux from ALEXI (Fig. 6) and LST (See Supplementary Fig. S4). Diagnostic modeling approaches such as ALEXI provide an estimate of energy balance elements; e.g. SH and LH fluxes without *a priori* specification of moisture inputs. ALEXI incorporates satellite observations into a model (see methodology for details) and provides an estimate which is a proxy for ground-truth. Atmospheric interference, particularly due to clouds, can lead to missing data and some noise in ALEXI. Persistent cloud-contamination results in missing data points in ALEXI, particularly during the rainy season. Hence, this diagnostic approach may not provide a smooth anomaly plot as in Figs 3 and 5. But Fig. 6 suggests that the anomalously high SH flux pattern spreads spatially in the weeks leading up to the heat wave, and this spread is geographically consistent with the COHW.

The local heating analysis is summarized in Table 1. In the late-May peak, clear skies led to enhanced longwave radiation that served to reinforce a heat wave that was primarily a product of large scale conditions. In June, dry conditions caused a substantial positive SH anomaly to emerge, resulting in a significant forcing on air temperature. This can be considered in terms of total heating potential over the course of each heat wave peak. If we take the extreme case of an air parcel that stays within the COHW and the planetary boundary layer for several days leading up to each heat wave peak then we can estimate the contribution of SH anomaly to the temperature anomaly of that parcel. This is an extreme end member. In fact, winds were light (Table 1) but of generally consistent direction (result not shown here), suggesting COHW residence times on the order of ~1 day for a parcel that transverses the core of the heat wave in the direction of prevailing winds. But the end member is instructive when comparing events. In the four days leading up to the high daily temperature anomaly in the late May peak on May 21st–22nd, the SAT anomaly rose by 4.5 °C, while the integrated SH anomaly would only create a heating on the order of 0.2 °C for the theoretical air parcel that stays within COHW_May_ for the full four days. For the June peak, in contrast, SAT anomaly rose by only 2.5 °C on June 9th-10th relative to the preceding period, but the integrated SH anomaly could warm a stagnant air parcel by ~4 °C. This points to the importance of the local energy partitioning anomaly due to soil moisture deficit during the June heat event.

## Conclusions

In this paper, we examined the spatial and temporal pattern of the 2015 India heat wave and quantified the potential for land surface conditions to contribute to the heat extreme. We have employed a suite of models and datasets to the analysis, including meteorological station observations, reanalysis output (primarily MLD), South Asia LDAS, a satellite based diagnostic model (ALEXI), and standard remote sensing products (MODIS).

We find that the heat wave struck India in two phases: first in late May and again in early June. Both phases were associated with low rainfall and unusually clear skies, leading to a positive anomaly in R_net_ at the surface and enhanced local heating from the land surface. This result complements the study^10^ which identified clear skies associated with large-scale atmospheric conditions as a driver of HW15. During the May phase of the heat wave, persistent dry atmospheric conditions and elevated incoming SW radiation cause a soil moisture deficit to develop. Thus, a soil moisture mediated energy partitioning feedback on temperature appears to lag the May heat wave peak but lead the temperature anomaly peak in June in the center of the heat wave. As a result, enhanced sensible heat flux associated with a dry surface contributed much more significantly to the June peak than it did to the late May peak. This conclusion is supported by satellite derived temperature and heat flux estimates, which show anomalously warm land surface temperature (MODIS) and anomalously high sensible heat flux (ALEXI) during the peak of heat wave.

These results demonstrate the potential for both large scale atmospheric dynamics and local feedbacks to contribute to pre-monsoon heat waves in India. For HW15, the relative contribution of each changed over the course of the event as land surface conditions evolved, with local heating becoming increasingly important in the second phase of the heat wave. As extreme heat is of increasing concern in India, and as the impact of climate change on the onset of monsoon rains is an area of significant uncertainty, understanding, monitoring, and, where possible, managing the impact that land surface conditions have on the development of extreme heat events should receive continued attention.

### Data and Methodology

HW15 is studied using a combination of atmospheric reanalysis data, land surface model simulations, and satellite-derived observations. We use surface state and near-surface meteorology fields drawn from the MERRA (Modern Era Retrospective Analysis for Research and Applications) -Land (MLD) data product^12^. MLD improves MERRA’s representation of the land surface in part by merging a gauge-based precipitation product from NOAA CPC with MERRA precipitation. For this study we make use of daily surface air temperature (SAT), total precipitation, net shortwave radiation and net longwave radiation from MLD. MLD estimates of SAT was compared to those of the ERA Interim reanalysis^13^ and were found to be similar (Fig. S3). MLD temperature estimates are used to define the Core of the Heat Wave (COHW), which is used as the basis for all area averaged calculations presented in the results section. We do note that there is heterogeneity within the COHW due to surface properties and local weather.

To address the biases present in the reananlysis product^14^, we complement the reanalysis dataset by analyzing *in situ* meteorological records from the National Climate Data Center archive WMO GSOD network, obtained from the NOAA National Climate Data Center (https://gis.ncdc.noaa.gov/maps/ncei/cdo/daily). In addition, we perform our own offline land surface model simulations to study details of land surface conditions up to and during HW15. A 36 year long simulation (1980–2015) was performed using Noah 3.3 land surface model^15^ under the South Asia Land Data Assimilation System (South Asia LDAS) framework^16^. The simulations were performed at 10 km resolution, had a 36 year spin-up, used MLD as meteorological forcing, used satellite-derived land cover and vegetation parameters, and accounted for irrigation. We use daily soil moisture and sensible heat flux outputs from the LDAS.

Finally, several satellite-derived datasets were used to provide an independent view of HW15. Moderate Resolution Imaging Spectroradiometer (MODIS) land surface temperature (LST) fields at 5 km horizontal resolution were used as a complementary temperature dataset (MOD11C2)^17^. The Atmosphere-Land Exchange Inverse Model (ALEXI)^18–21^ estimates of surface sensible heat flux are also applied. ALEXI derives surface turbulent heat flux estimates on the basis of a two-source land surface model coupled with a one-dimensional atmospheric boundary layer model. The version of ALEXI used in this study applies time-differential measurements of morning land surface temperature rise to diagnose the partitioning of available energy into sensible, latent, and ground heat flux components^21^.

## Electronic supplementary material

Supplementary Information

## References

[1] Jenamani RK (2012). Analysis of Ocean-Atmospheric features associated with extreme temperature variations over east coast of India- A special emphasis to Orissa heat waves of 1998 and 2005. Mausam..

[2] Bhadram CVV, Amatya BVS, Pant GB, Kumar KK (2005). Heat waves over Andhra Pradesh: A case study of summer of 2003. Mausam..

[3] Pattanaik DR, Hatwar HR (2006). Analysis and impact of delayed onset of monsoon over Northeast India during 2005. Vayu Mandal..

[4] Pattanaik, D. R., Mohapatra, M., Srivastava, A. K. & Kumar A. Heat wave over India during summer 2015: an assessment of real time extended range forecast. *Meteorology and Atmospheric Physics*. 1–19 (2016).

[5] Murari KK, Ghosh S, Patwardhan A, Daly E, Salvi K (2015). Intensification of future severe heat waves in India and their effect on heat stress and mortality. Reg. Environ Change..

[6] Fischer EM, Seneviratne SI, Vidale PL, Lüthi D, Schär C (2007). Soil Moisture–Atmosphere Interactions during the 2003 European Summer Heat Wave. J. Climate..

[7] Zaitchik BF, Macalady AK, Bonneau LR, Smith RB (2006). Europe’s 2003 heat wave: a satellite view of impacts and land–atmosphere feedbacks. Int. J. Climatol..

[8] Miralles DG, Teuling AJ, van Heerwaarden CC, de Arellano VG (2014). Mega-heatwave temperatures due to combined soil desiccation and atmospheric heat accumulation. Nat. Geosci...

[9] Rohini P, Rajeevan M, Srivastava AK (2016). On the Variability and Increasing Trends of Heat Waves over India. Scientific Reports..

[10] Ratnam JV, Behera SK, Ratna SB, Rajeevan M, Yamagata T (2016). Anatomy of Indian heatwaves. Scientific Reports..

[11] Murari KK, Sahana AS, Daly E, Ghosh S (2016). The influence of the El Niño Southern Oscillation on heat waves in India. Met. Apps..

[12] Reichle RH (2011). Assessment and enhancement of MERRA land surface hydrology estimates. Journal of Climate..

[13] Dee DP (2011). The ERA-Interim reanalysis: configuration and performance of the data assimilation system. Q.J.R. Meteorol. Soc..

[14] Shah R, Mishra V (2014). Evaluation of the Reanalysis Products for the Monsoon Season Droughts in India. J. Hydrometeor..

[15] Ek MB (2003). Implementation of Noah land surface model advances in the National Centers for Environmental Prediction operational mesoscale Eta model. J. Geophys. Res..

[16] Ghatak, D. *et al*. Characterizing hydrological hazards and trends with the NASA South Asia Land Data Assimilation System. American Geophysical Union Fall Meeting. San Francisco, USA. Dec 2015.

[17] Wan Z, Zhang Y, Zhang Q, Li ZL (2002). Validation of the land-surface temperature products retrieved from Terra Moderate Resolution Imaging Spectroradiometer data. Remote Sens. Environ..

[18] Anderson MC, Norman JM, Diak GR, Kustas WP, Mecikalski JR (1997). A two-source time-integrated model for estimating surface fluxes using thermal infrared remote sensing. Remote Sens. Environ..

[19] Anderson, M. C., Kustas, W. P. & Norman, J. M. Upscaling tower and aircraft fluxes from local to continental scales using thermal remote sensing. *Agron. J.* **99**, 240–254, doi:10.2134/agronj2005.0096S (2007a).

[20] Anderson MC (2007). A climatological study of evapotranspiration andmoisture stress across the continental United States: 1. Model formulation. J.Geophys. Res..

[21] Hain CR, Crow WT, Anderson MC, Yilmaz MT (2015). Diagnosing Neglected Soil Moisture Source–Sink Processes via a Thermal Infrared–Based Two-Source Energy Balance. Model. J. Hydrometeor..

