# Supplementary material for: The role of local heating in the 2015 Indian Heat Wave
Source: Sci Rep. 2017 Aug 9;7:7707. doi: 10.1038/s41598-017-07956-5 (PMC5550505; doi:10.1038/s41598-017-07956-5)
Supplement: Supplementary file 1 — Supplementary Information [file 41598_2017_7956_MOESM1_ESM.pdf]

Title: The role of local heating in the 2015 Indian Heat Wave

Debjani Ghatak[1], Benjamin Zaitchik[2] , Christopher Hain[3] , Martha Anderson[4]

1 dghatak1@jhu.edu, Department of Earth and Planetary Sciences, Johns Hopkins University, Baltimore, Maryland

2 zaitchik@jhu.edu, Department of Earth and Planetary Sciences, Johns Hopkins University, Baltimore, Maryland

3 christopher.hain@nasa.gov, NASA Marshall Space Flight Center, Earth Science Branch, Huntsville, Alabama

4 martha.anderson@ars.usda.gov, Hydrology and Remote Sensing Laboratory, Agricultural Research Service, USDA, Beltsville, Maryland

Supplementary Figure Caption:

Figure S1. Grey shade shows the region where MLD SAT for the week of May 21st-22nd to May 27th-28th meets or exceeds the 90th percentile of the average SAT for the corresponding week. Data visualizations produced using IDL [8.4] (Exelis Visual Information Solutions, Boulder, Colorado).

Figure S2. Comparison between daily SAT (°C) from MLD output (blue box plots) and from observations (green box plots) for multiple stations in and around the COHW. Time domain is 1990-2015 except for some stations (Daltonganj, Hyderabad, PBO Raipur and Varanasi) where observations were limited.

Figure S3: SAT anomaly (°C) for the month of May 2015 based on the monthly climatology of 1980-2015 based on (a) MLD and (b) ERA-I reanalyses. Data visualizations produced using IDL [8.4] (Exelis Visual Information Solutions, Boulder, Colorado).

Figure S4. MODIS LST 8-day composite anomaly (°C) based on 8-day climatology for the period of 2003-2015 for (a) May 17th-24th (b) May 25th–June1st and (c) June 2nd-9th. Any pixel with elevation above 1000m is not shown (white colored region). White color also shows pixel with missing values. Data visualizations produced using IDL [8.4] (Exelis Visual Information Solutions, Boulder, Colorado).

Supplementary Table Caption:

Table S1: Geographical locations of the stations and Pearson correlation coefficient between station and MLD SAT timeseries for the period of May-June in 2015, as shown in Figure 1

Table S2: Pearson correlation coefficient between daily station and MLD SAT timeseries for the period 1990-2015. For certain stations (Daltonganj, Hyderabad, PBO Raipur and Varanasi) the period of record is limited due to data availability.

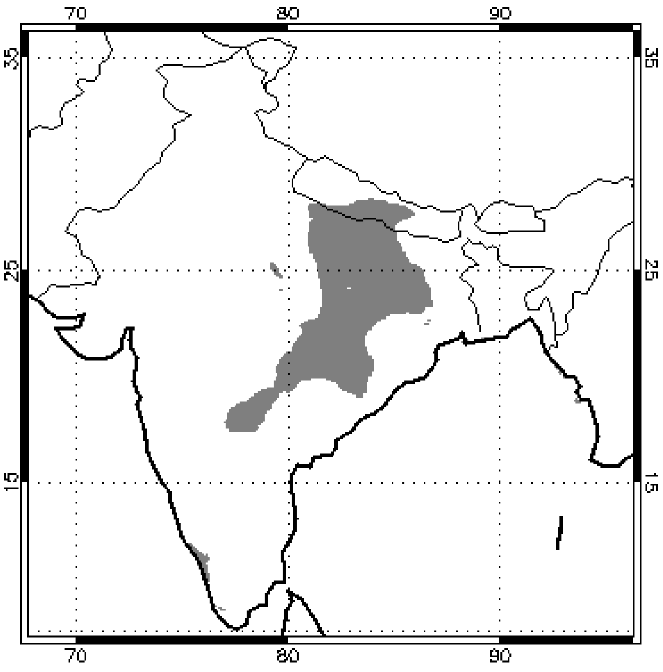

Figure S1. Grey shade shows the region where MLD SAT for the week of May 21st-22nd to May 27th-28th meets or exceeds the 90th percentile of the average SAT for the corresponding week. Data visualizations produced using IDL [8.4] (Exelis Visual Information Solutions, Boulder, Colorado).

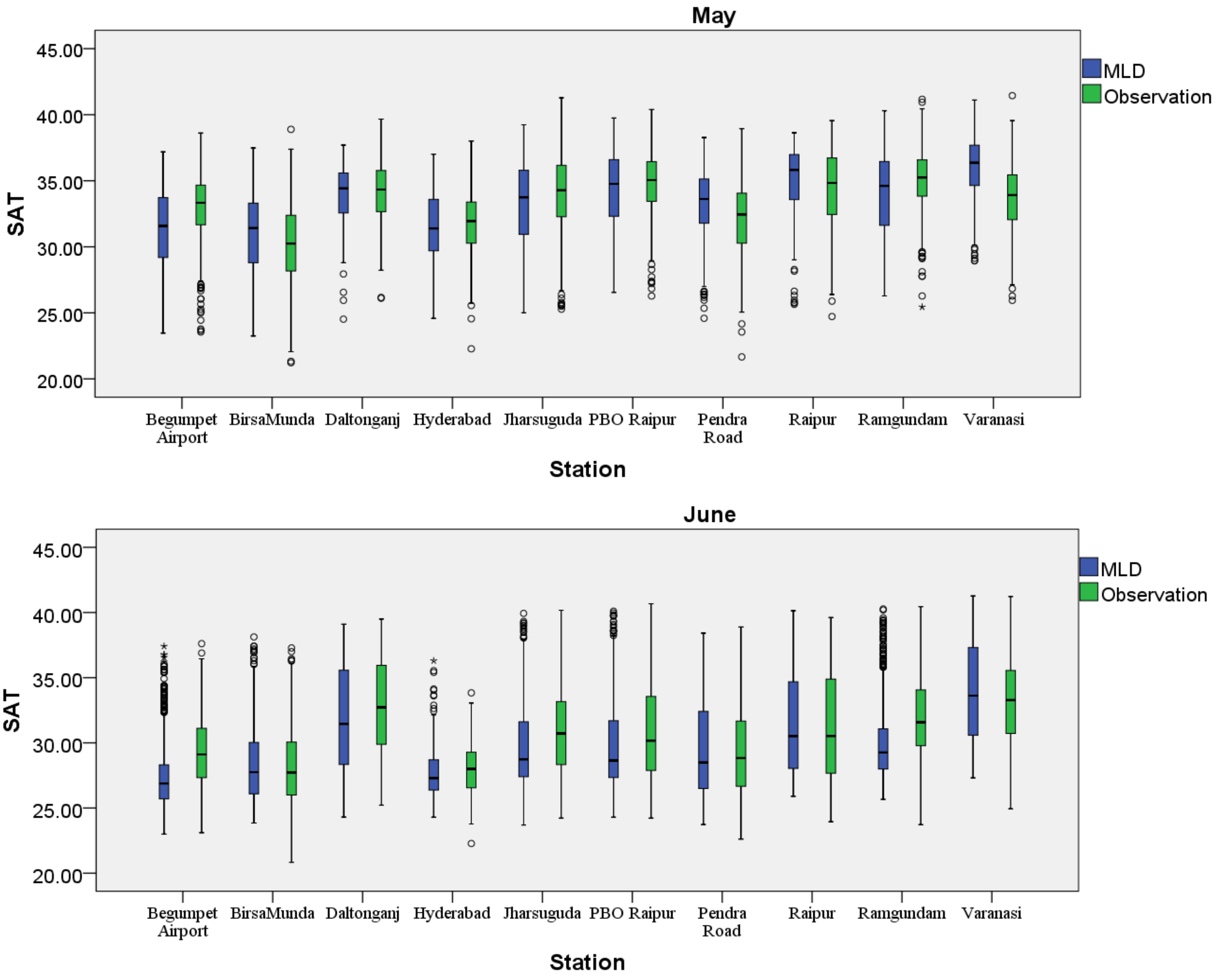

Figure S2. Comparison between daily SAT (°C) from MLD output (blue box plots) and from observations (green box plots) for multiple stations in and around the COHW. Time domain is 1990-2015 except for some stations (Daltonganj, Hyderabad, PBO Raipur and Varanasi) where observations were limited. Data visualizations produced using IBM SPSS software version 23.

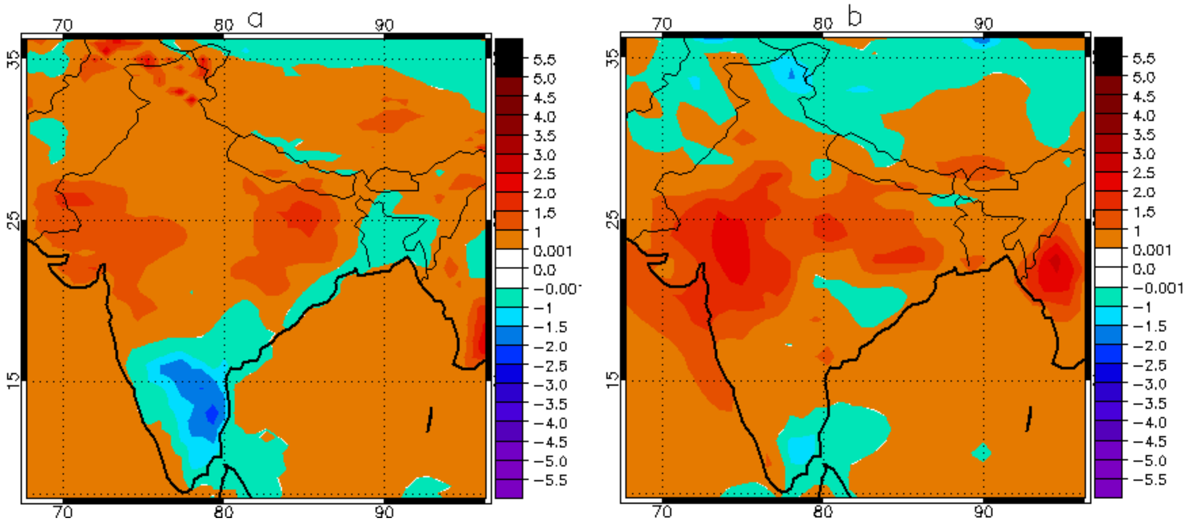

Figure S3: SAT anomaly (°C) for the month of May 2015 based on the monthly climatology of 1980-2015 based on (a) MLD and (b) ERA-I reanalyses. Data visualizations produced using IDL [8.4] (Exelis Visual Information Solutions, Boulder, Colorado).

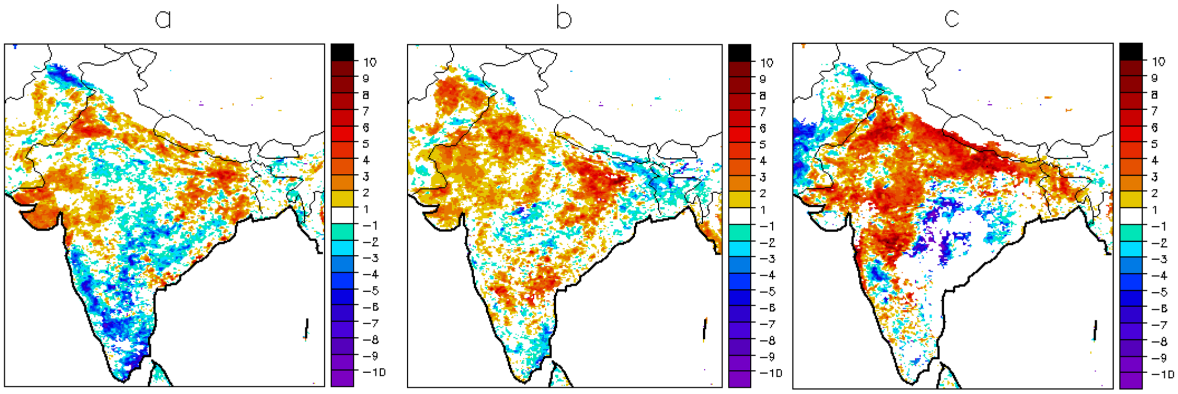

Figure S4. MODIS LST 8-day composite anomaly (°C) based on 8-day climatology for the period of 2003-2015 for (a) May 17th-24th (b) May 25th–June1st and (c) June 2nd-9th. Any pixel with elevation above 1000m is not shown (white colored region). White color also shows pixel with missing values. Data visualizations produced using IDL [8.4] (Exelis Visual Information Solutions, Boulder, Colorado).

Table S1: Geographical locations of the stations and Pearson correlation coefficient between station and MLD SAT timeseries for the period of May-June in 2015, as shown in figure 1

	**Goya**	**Daltonganj**	**Jharsuguda**	**Jabalpur**	**Ramgundam**	**Begumpet Airport**
Latitude and Longitude	+24.744<br>+84.951	+24.050<br>+84.067	+21.914<br>+84.050	+23.178<br>+80.052	+18.767<br>+79.433	+17.452<br>+78.461
R value	0.8	0.84	0.78	0.83	0.88	0.89

Table S2: Pearson correlation coefficient between daily station and MLD SAT timeseries for the period 1990-2015. For certain stations (Daltonganj, Hyderabad, PBO Raipur and Varanasi) the period of record is limited due to data availability.

Station Name	May	June
Begumpet (+17.452, +78.461)	0.66	0.70
BirsaMunda (+23.314, +85.322)	0.70	0.79
Daltonganj (+24.05 , +84.067)	0.63	0.85
Hyderabad (+25.383, +68.417)	0.67	0.71
Jharsuguda (+21.914, +84.05)	0.68	0.82
PBO_Raipur (+21.233, +81.65)	0.65	0.82
PendraRoad (+22.767, +81.9)	0.68	0.83
Raipur (+21.18, +81.739)	0.73	0.86
Ramagundam (+18.767 ,+79.433)	0.62	0.72
Varanasi (+25.452, +82.859)	0.6	0.74
